# Supplementary material for: Anaerobic photoinduced Cu(0/I)-mediated Glaser coupling in a radical pathway
Source: Nat Commun. 2023 Oct 24;14:6741. doi: 10.1038/s41467-023-42602-x (PMC10598264; doi:10.1038/s41467-023-42602-x)
Supplement: Supplementary file 1 — Supplementary Information [file 41467_2023_42602_MOESM1_ESM.pdf]

Supplementary Information

Anaerobic photoinduced Cu(0/I)-mediated Glaser coupling in a radical pathway

Siqi Zhang[1,2] & Liang Zhao[1]*

[1]Key Laboratory of Bioorganic Phosphorus Chemistry & Chemical Biology (Ministry of Education), Department of Chemistry, Tsinghua University, Beijing 100084, China.

[2]Jiangsu Key Laboratory of New Drug Research and Clinical Pharmacy, Xuzhou Medical University, Xuzhou, Jiangsu 221004, China.

E-mail: zhaolchem@mail.tsinghua.edu.cn; Phone: +86-10-62786635.

The authors declare no conflict of interest.

1. X-ray crystallographic studies

Single-crystal X-ray data for Cu-O complexes were collected with Cu-Kα radiation (λ = 1.54184 Å) on a Rigaku Saturn 724/724 + CCD diffractometer with frames of oscillation range 0.5°. The selected crystal was mounted onto a nylon loop by polyisobutene and enveloped in a low-temperature stream of dry nitrogen gas during data collection. The absorption corrections were applied using multi-scan methods. All structures were solved using the SHELXT program, and by using Olex2 (Dolomanov et al., 2009) as the graphical interface. The models were refined with ShelXT 2018/3 (Sheldrick, 2018) using full-matrix least-squares on F^2. All figures were drawn by using X-seed program.

Crystal data for Complex 1 ($[Cu_8(\mu_3\text{-}PhC{\equiv}C)_4(CH_3CN)_4(Py[8])](BF_4)_4{\cdot}3(CH_3OH){\cdot}(H_2O)$) (CCDC-2252404): $C_{97}H_{86}B_4C_{l2}Cu_8F_{16}N_{20}O$, M = 2474.31, monoclinic, space group $P2_1/c$, a = 17.4859(3) Å, b = 34.8145(8) Å, c = 18.2949(5) Å, α = 90°, β = 112.612(3)°, γ = 90°, V = 10281.1(5) $Å^3$, Z = 4, T = 100 K, Dc = 1.599 g cm^{-3}. The structure, refined on F^2, converged for 20301 unique reflections (R_{int} = 0.1493) and 15843 observed reflections with I > 2σ(I) to give R_1 = 0.1085 and wR_2 = 0.3139 and a goodness-of-fit = 1.051. One fluorine atom of BF_4 anion is disordered at two separated positions with a refined occupancy ratio of 0.75:0.25.

Crystal data for Complex 2 ($[Cu_3(\mu_2\text{-}PhC{\equiv}C)(\mu_2\text{-}O)(CH_3CN)(Py[8])](BF_4)_2{\cdot}(CH_2Cl_2)$) (CCDC-2252405): $C_{60.50}H_{53}B_2Cl_2Cu_3F_8N_{17}O$, M = 1469.34, triclinic, space group P-1, a = 12.0066(5) Å, b = 15.7006(7) Å, c = 18.8263(6) Å, α = 89.198(3)°, β = 76.977(3)°, γ = 68.061(4)°, V = 3197.6(3) $Å^3$, Z = 2, T = 105 K, Dc = 1.526 g cm^{-3}. The structure, refined on F^2, converged for 12078 unique reflections (R_{int} = 0.1256) and 7579 observed reflections with I > 2σ(I) to give R_1 = 0.1181 and wR_2 = 0.3723 and a goodness-of-fit = 1.213. Two fluorine atoms of one BF_4 anion are disordered at two separated positions with a refined occupancy ratio of 0.75:0.25. One chlorine atom of CH_2Cl_2 is disordered at two separated positions with a refined occupancy ratio of 0.50:0.50.

2. Supplementary figures

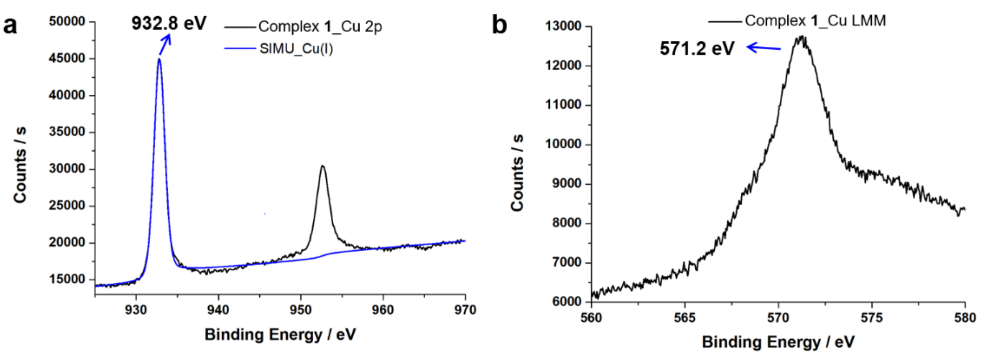

E_k = hv– E_b – Φ = hv – kE_b = 1486.6 eV – 571.2 eV = 915.4 eV
Auger parameter: E_k + E_b = 915.4 eV + 932.8 eV = 1848.2 eV **(CuCl: 1848.0 eV)**

Supplementary Figure 1. XPS spectra of complex 1. a, Cu 2p and **b**, Cu LMM spectra for the XPS of **1**.

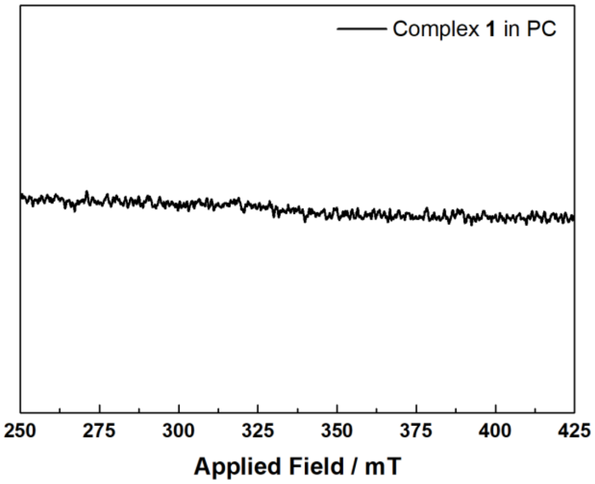

Supplementary Figure 2. EPR spectrum of complex 1. EPR spectrum of **1** at 130 K in propylene carbonate (PC, 9.44 GHz).

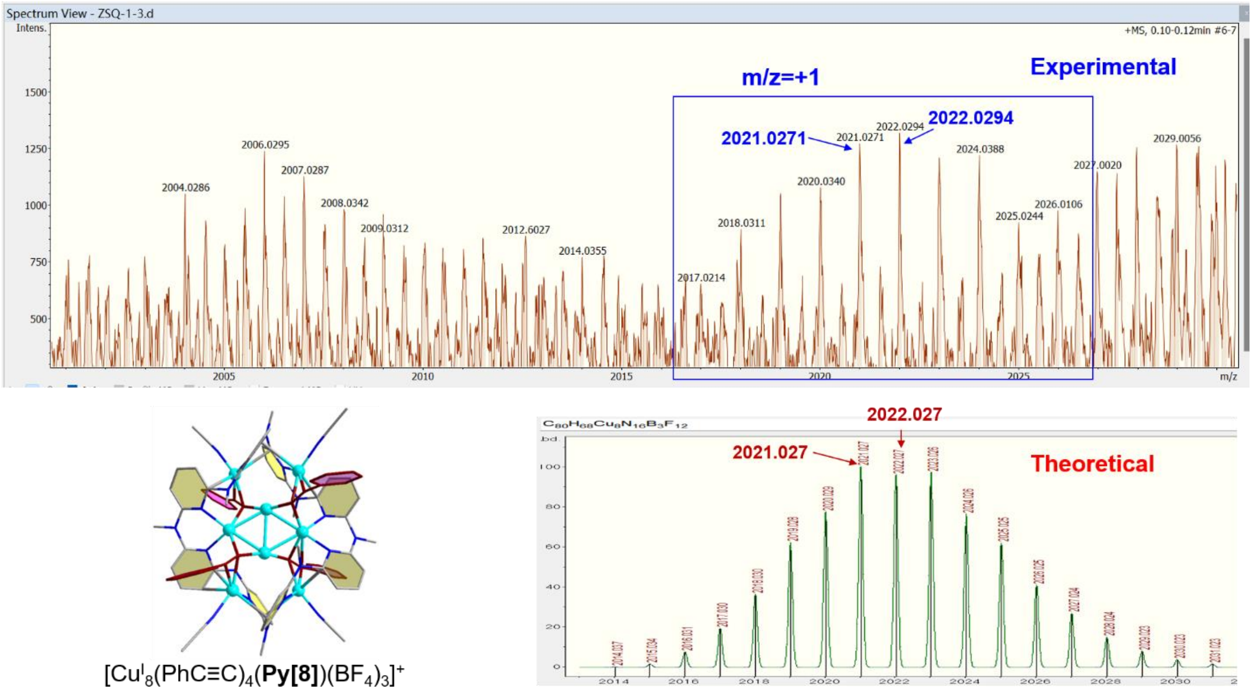

Supplementary Figure 3. High resolution ESI-MS of 1. High resolution ESI-MS of **1** in PC.

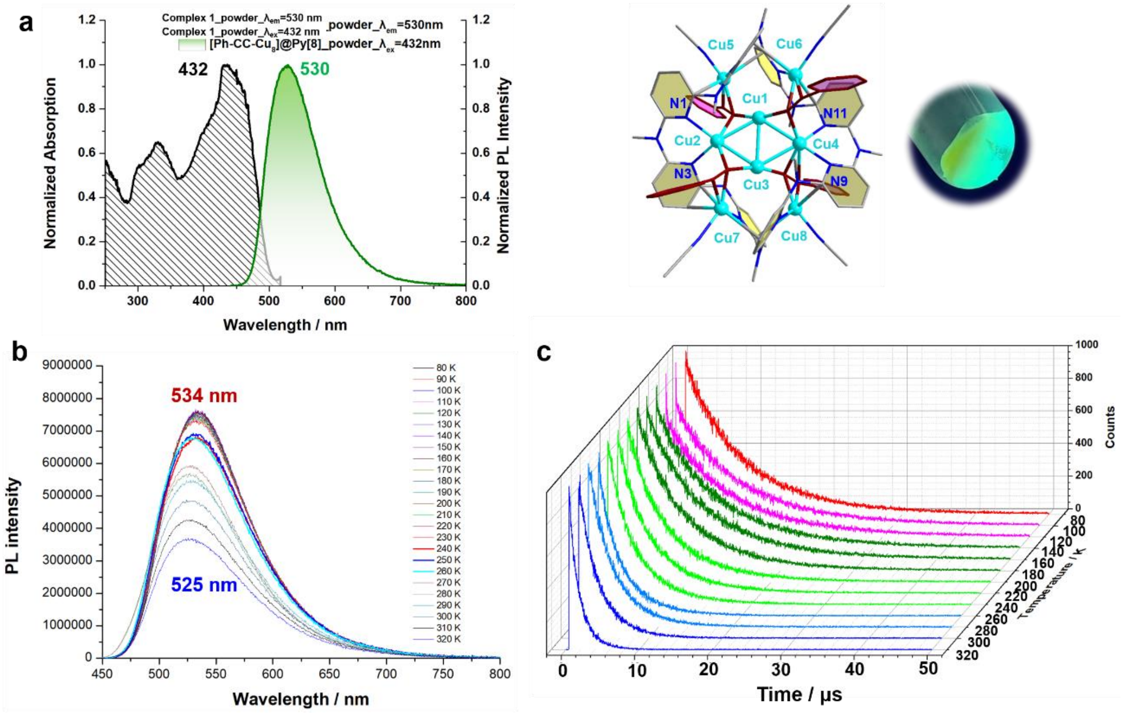

Supplementary Figure 4. Absorption and emission property of 1. a, Absorption and emission spectra of **1** in solid state. **b**, Photoluminescent intensity and **c**, lifetime of **1** in propylene carbonate (PC) under N_2 (80 K~320 K).

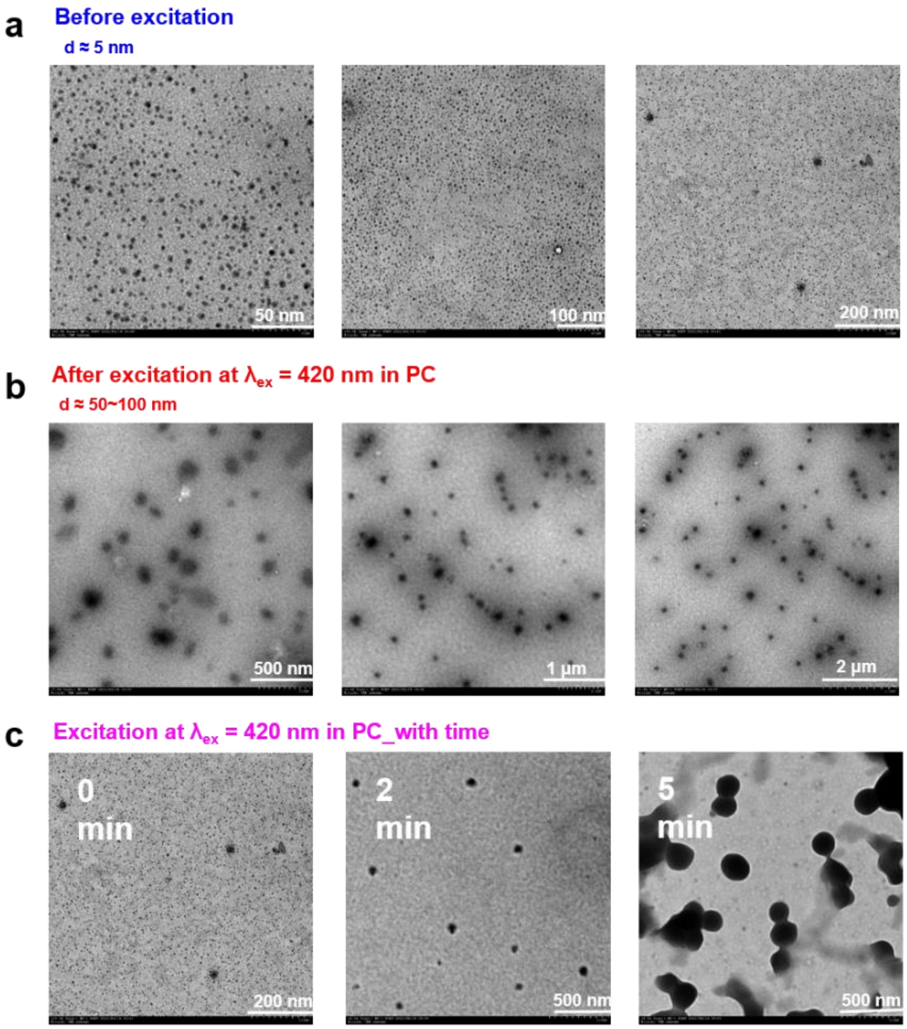

Supplementary Figure 5. TEM of 1. TEM of **1** in PC **a**, before and **b**, after excitation at 420 nm for 10 mins in N_2 atmosphere. **c**, TEM of **1** at λ_{ex} = 420 nm in N_2 atmosphere with different time intervals.

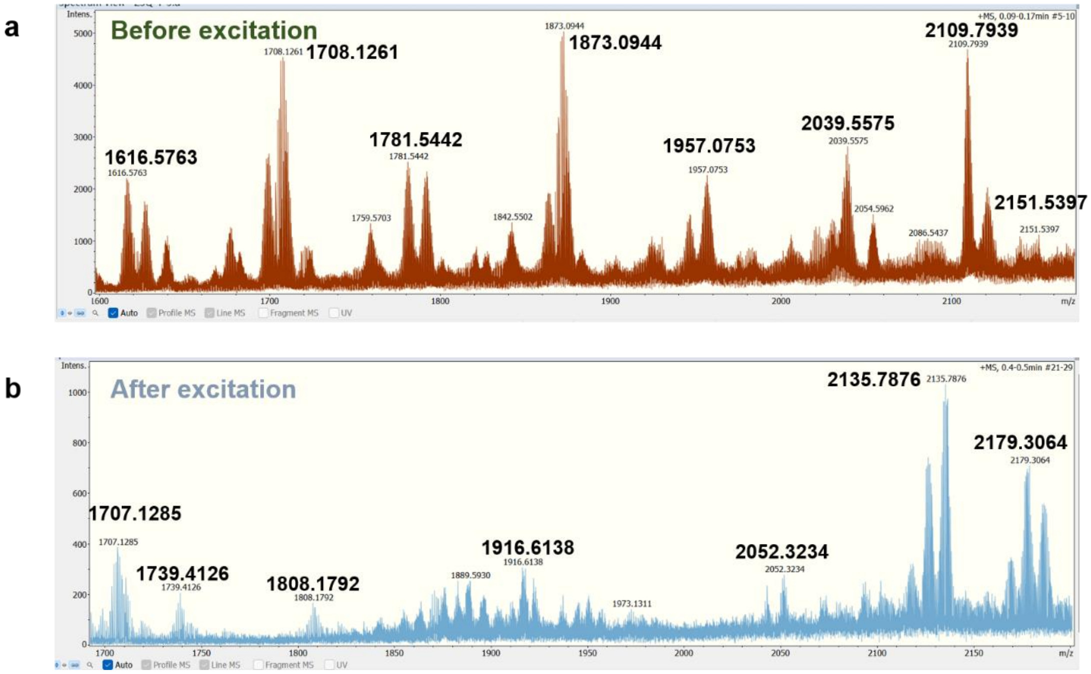

Supplementary Figure 6. HR-MS spectra. HR-MS of **1** in PC **a**, before and **b**, after excitation at 420 nm for 10 mins in N_2 atmosphere.

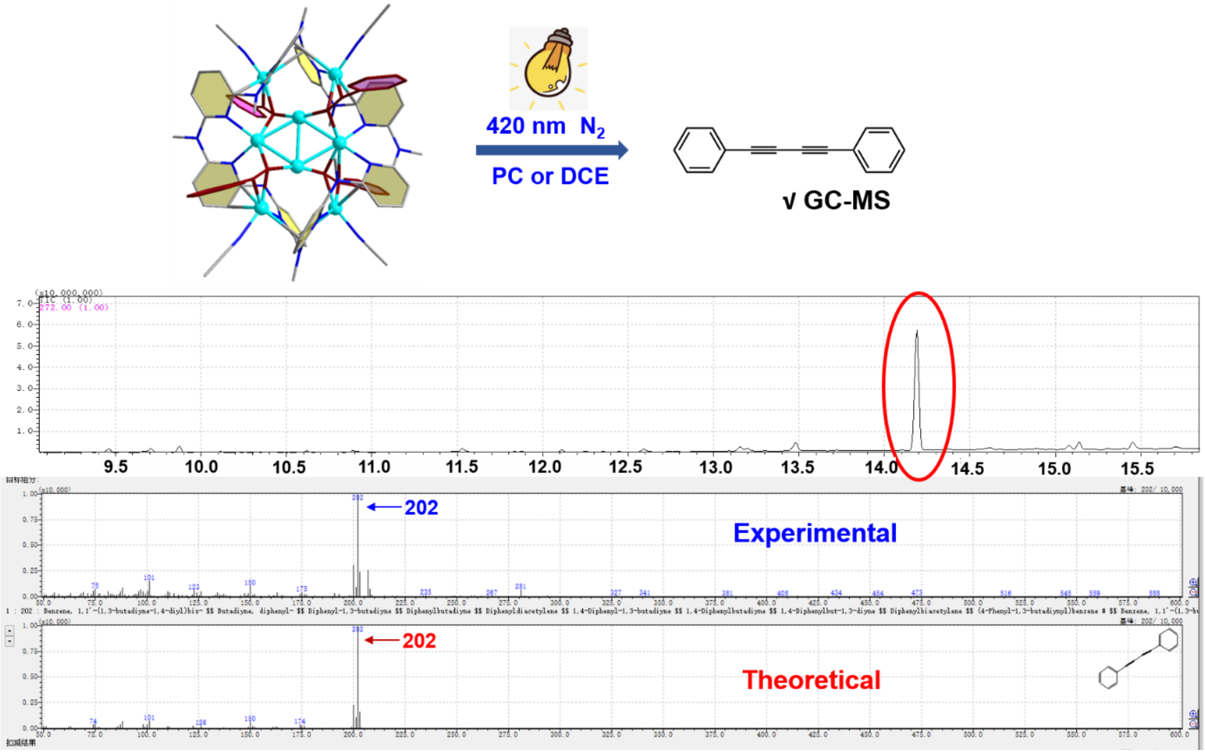

Supplementary Figure 7. GC-MS characterization. GC-MS for identifying the Glaser coupling product PhC≡C-C≡CPh when **1** in PC or dichloroethane (DCE) was irradiated at 420 nm for 10 mins in N_2 atmosphere.

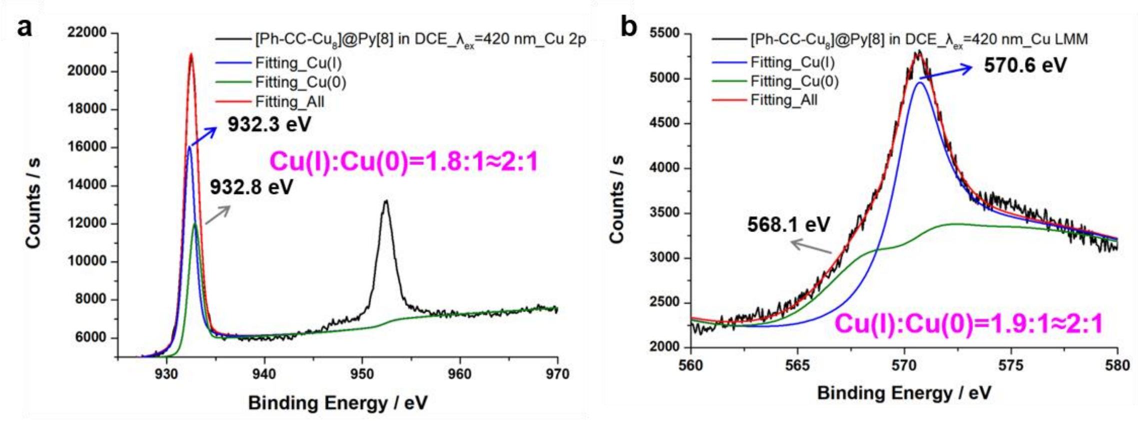

$E_k = h\nu - E_b - \Phi = h\nu - kE_b = 1486.6\ \text{eV} - 570.6\ \text{eV} = 916.0\ \text{eV}$

Auger parameter: $E_k + E_b = 916.0\ \text{eV} + 932.3\ \text{eV} = 1848.3\ \text{eV}$ **(CuCl: 1848.0 eV)**

$E_k = h\nu - E_b - \Phi = h\nu - kE_b = 1486.6\ \text{eV} - 568.1\ \text{eV} = 918.5\ \text{eV}$

Auger parameter: $E_k + E_b = 918.5\ \text{eV} + 932.8\ \text{eV} = 1851.3\ \text{eV}$ **(Cu: 1851.3 eV)**

➢ **No Cu(II) species after excitation**

Supplementary Figure 8. XPS spectra of 1 after excitation. **a**, Cu 2p and **b**, Cu LMM spectra for the XPS of **1** in DCE after excitation at 420 nm for 10 mins in N_2 atmosphere. The products were separated and purified by diethyl ether.

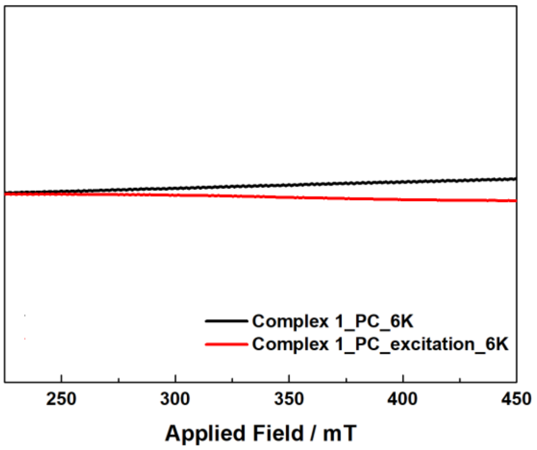

Supplementary Figure 9. EPR spectra. EPR of **1** in PC before (black) and after (red) excitation at 420 nm for 10 min in N_2 atmosphere.

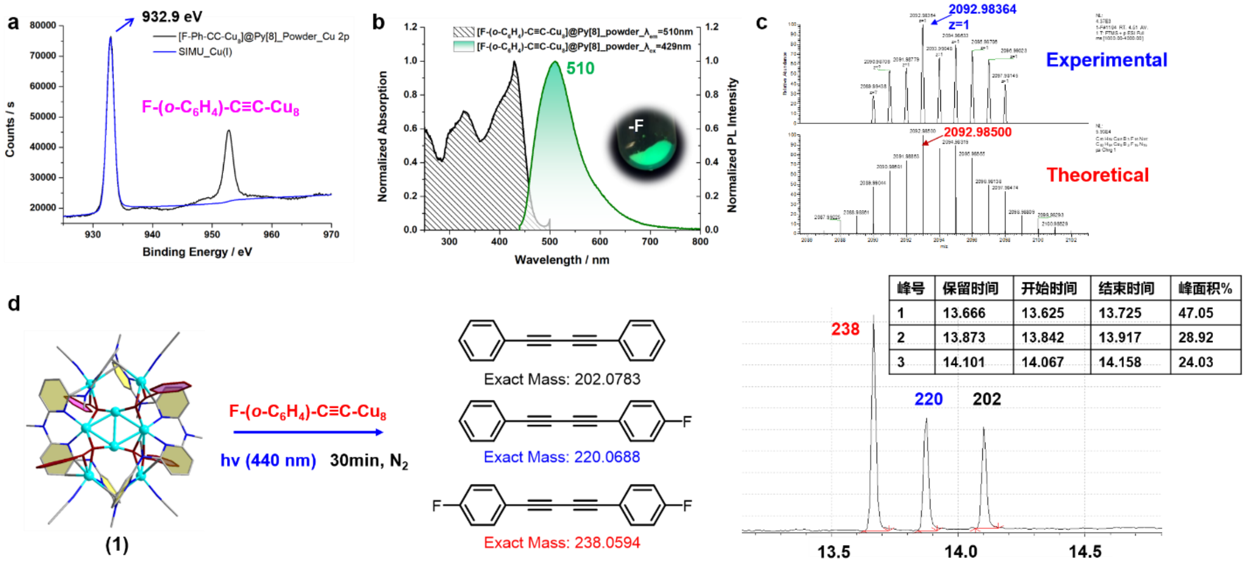

Supplementary Figure 10. Characterizations for 1-F. a, XPS, **b**, absorption and PL intensity spectra and **c**, HR-MS of the F-substituted analogue $[(4\text{-F-C}_6\text{H}_4\text{-C}\equiv\text{C})_4\text{Cu}^{\text{I}}_8(\text{MeCN})_4]$@**Py[8]** (**1-F**). **d**, Cross reaction between **1** and **1-F** in PC under the irradiation at 420 nm in N_2 atmosphere for 30 min, and the corresponding GC-MS spectra.

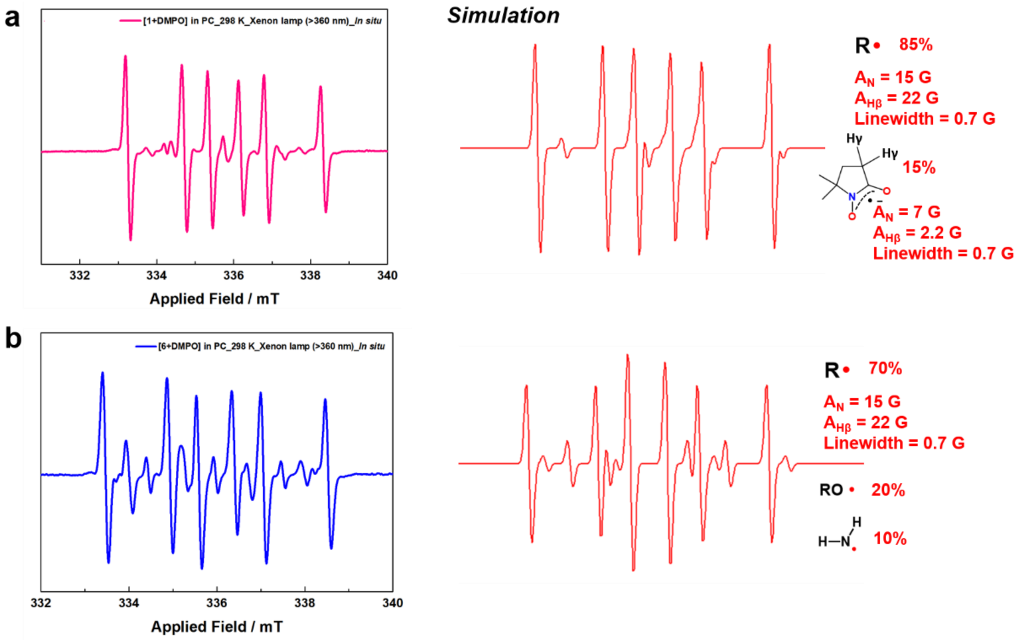

Supplementary Figure 11. EPR spin trapping experiments and simulations. EPR spin trapping experiments with DMPO for **a**, **1** and **b**, **6** in PC under *in situ* irradiation with Xenon lamp (filter > 360 nm) in N_2 atmosphere. The RO• and H_2N• result from the residual EtOH and NH_3H_2O during the synthesis of **6**.

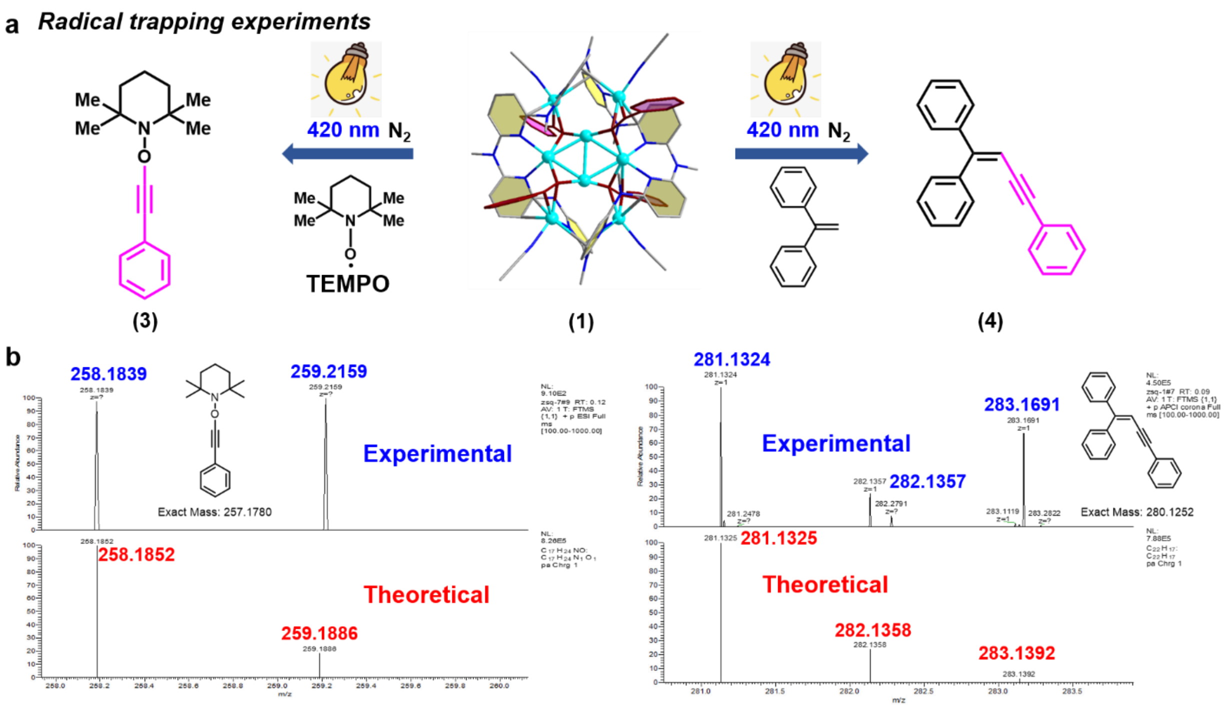

Supplementary Figure 12. Radical trapping experiments for 1. a, Radical trapping experiments in PC (10 mins, N_2 atmosphere) and **b**, HR-MS for the corresponding products **3** and **4**.

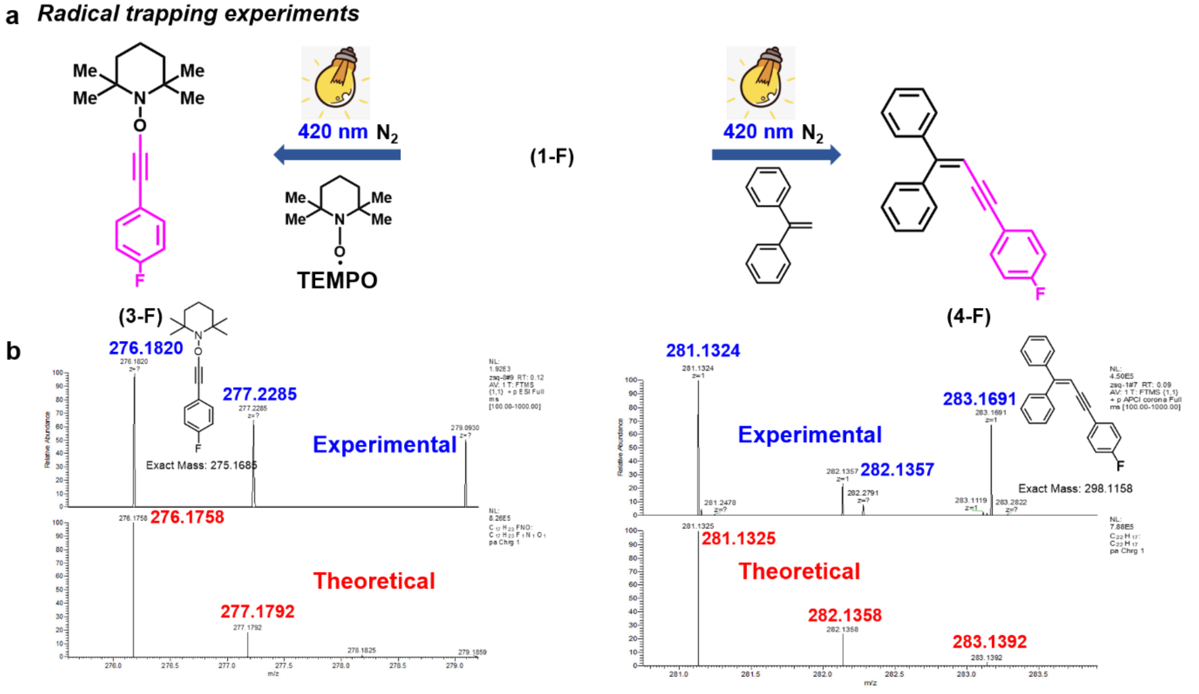

Supplementary Figure 13. Radical trapping experiments for 1-F. a, Radical trapping experiments in PC (10 mins, N_2 atmosphere) and **b**, HR-MS for the corresponding products **3-F** and **4-F**.

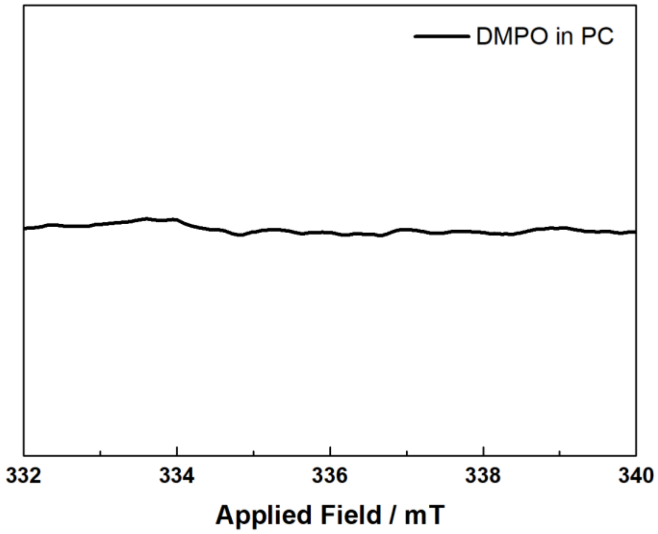

Supplementary Figure 14. EPR for DMPO. EPR spectra of DMPO in PC (N_2 atmosphere).

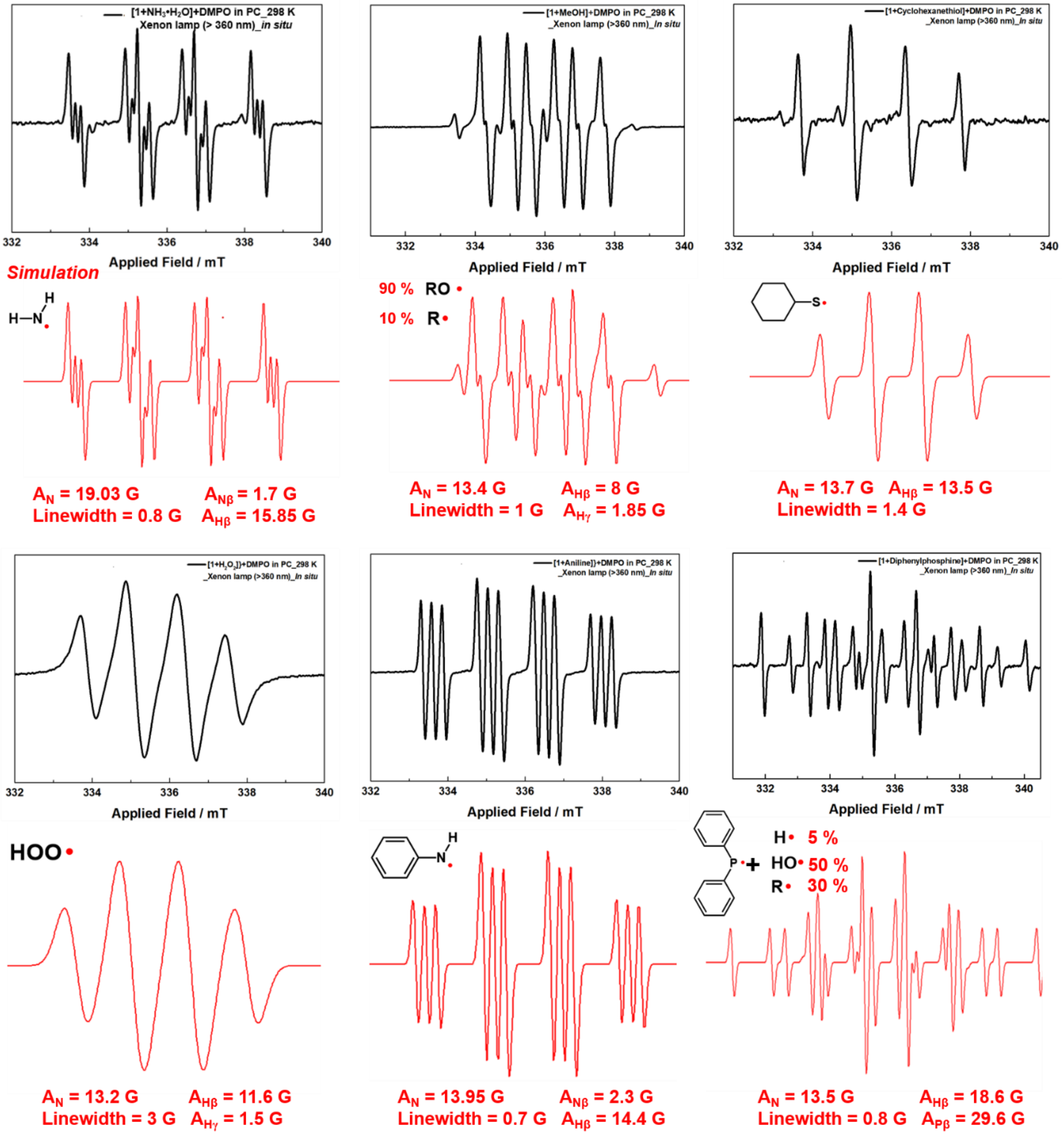

Supplementary Figure 15. EPR spin trapping experiments and simulations. EPR spin trapping experiments with DMPO for [**1**+X-H] in PC under *in situ* irradiation with Xenon lamp (filter > 360 nm) in N_2 atmosphere.

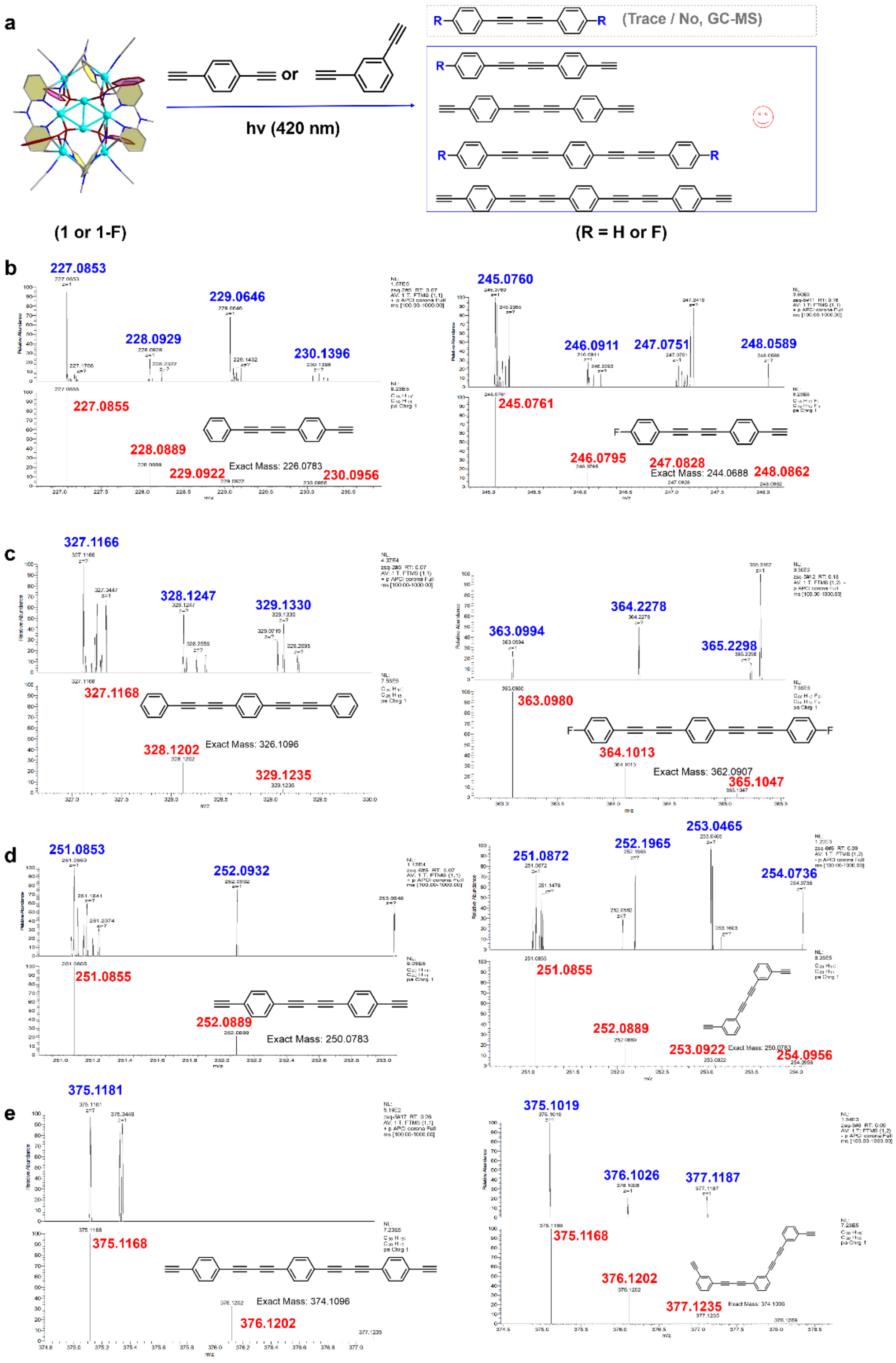

Supplementary Figure 16. HR-MS for products. a, Reaction for **1** or **1-F** with diynes. **b-e,** HR-MS for the oligomerized products in the addition of diynes in PC under the irradiation at 420 nm in N_2 atmosphere for 30 min.

EPR spin-trapping experiments

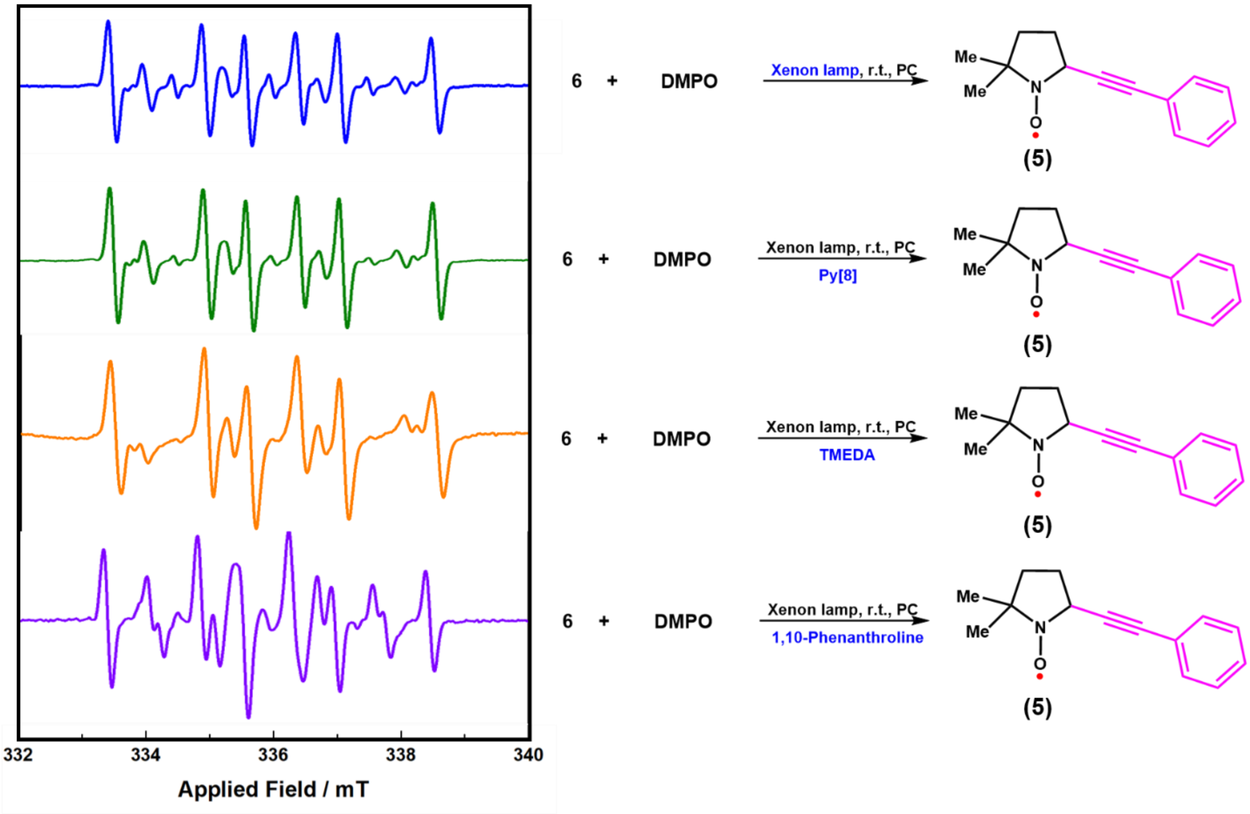

Supplementary Figure 17. EPR trapping with DMPO for [6+L]. EPR spin trapping experiments with DMPO for [**6**+**L**] (**L** = **Py[8]**, TMEDA or 1,10-phenanthroline) **in PC** under ***in situ*** irradiation with Xenon lamp (filter > 360 nm) in N_2 atmosphere.

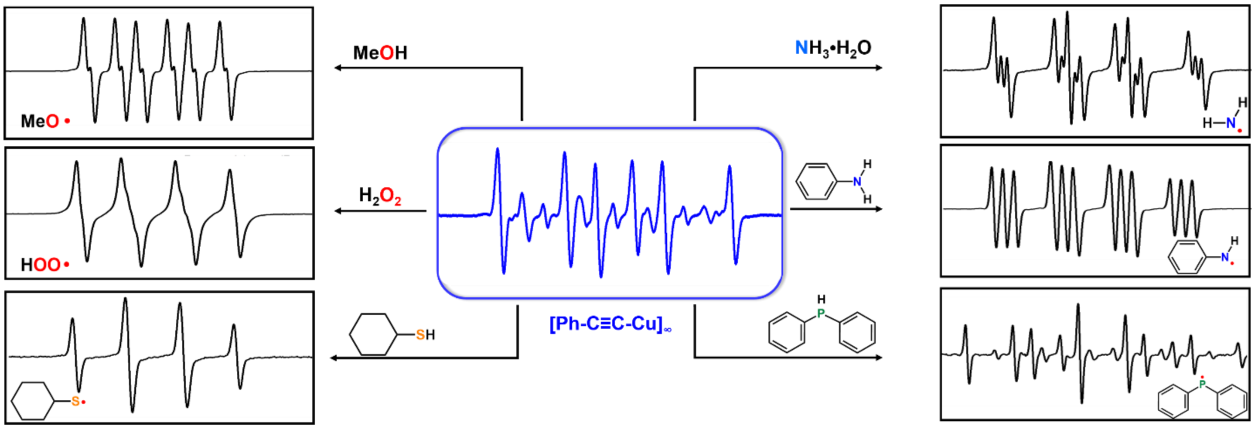

Supplementary Figure 18. EPR trapping with DMPO for [6+X-H]. EPR spin trapping experiments with DMPO for [**6**+X-H] (X = O, N, S, P) in PC under *in situ* irradiation with Xenon lamp (filter > 360 nm) in N_2 atmosphere.

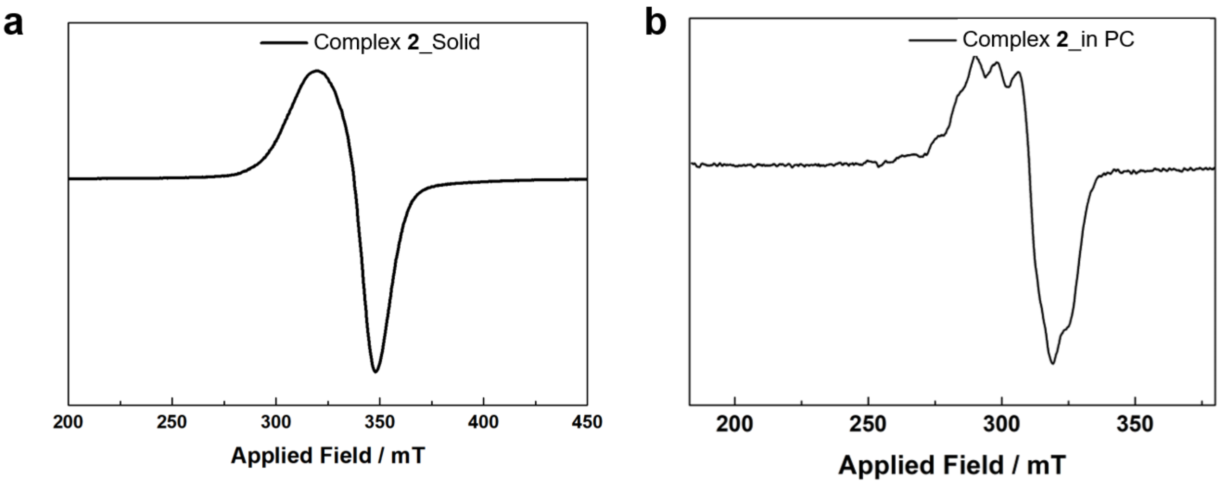

Supplementary Figure 19. EPR characterization of 2. EPR spectra of **2 a**, in solid state and **b**, in propylene carbonate (130 K, 9.04 GHz).

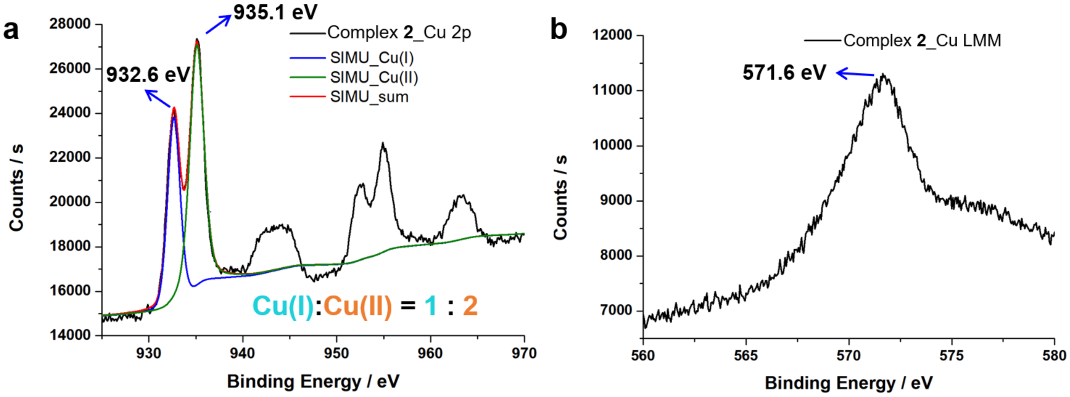

$E_k = hv – E_b – \Phi = hv – kE_b$ = 1486.6 eV – 571.6 eV = 915.0 eV
Auger parameter: $E_k + E_b$ = 915.0 eV + 932.6 eV = 1847.6 eV **(CuCl: 1847.6 eV)**

$E_k = hv – E_b – \Phi = hv – kE_b$ = 1486.6 eV – 571.6 eV = 915.0 eV
Auger parameter: $E_k + E_b$ = 915.0 eV + 935.1 eV = 1850.1 eV **($CuCl_2$: 1849.9 eV)**

Supplementary Figure 20. XPS spectra of 2. a, Cu 2p and **b**, Cu LMM spectra for the XPS of **2**.

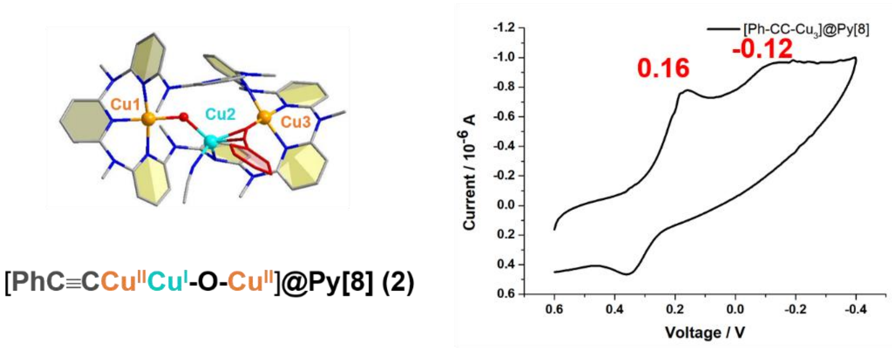

Supplementary Figure 21. CV characterization for 2. CV for **2** (0.5 mM) in deaerated PC (0.1 M Bu_4NPF_6) at 298 K with a glassy carbon working electrode and a Ag/AgCl reference electrode. Scan rate: 50 mV s^{-1}.

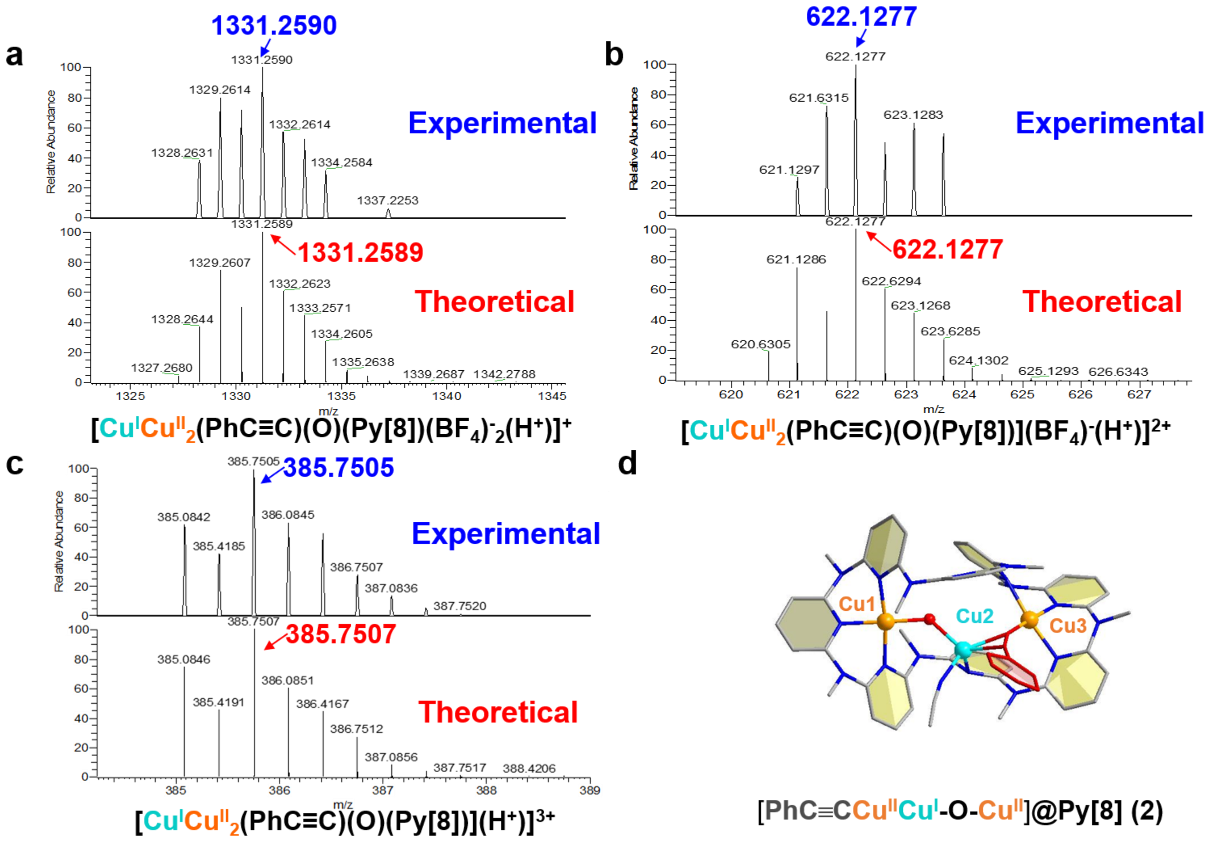

Supplementary Figure 22. HR-MS characterization for 2. a-c, High resolution ESI-MS spectra of **2** in PC. **d**, Crystal structure of **2**.

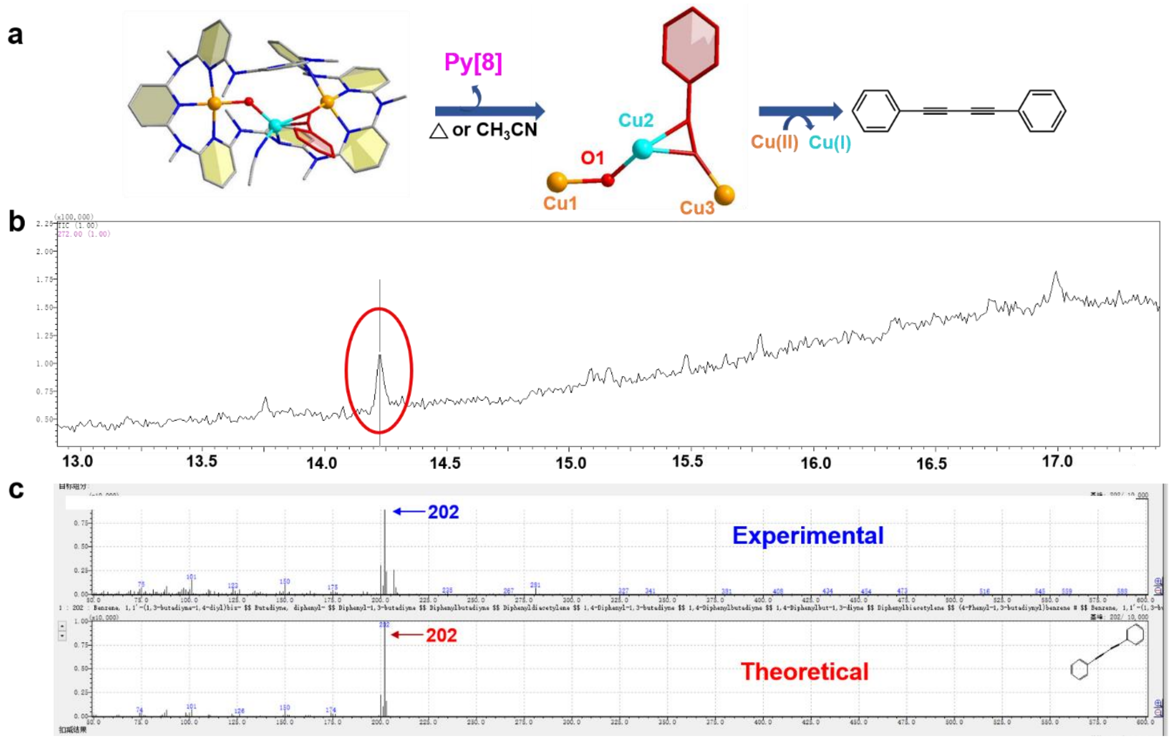

Supplementary Figure 23. GC-MS for PhC≡C-C≡CPh from 2. a, Release of the cluster core from **Py[8]** in **2** and further coupling reaction. **b-c**, GC-MS for identifying the Glaser coupling product PhC≡C-C≡CPh when **2** in PC was heated or added with a coordinative solvent (e.g. CH_3CN).

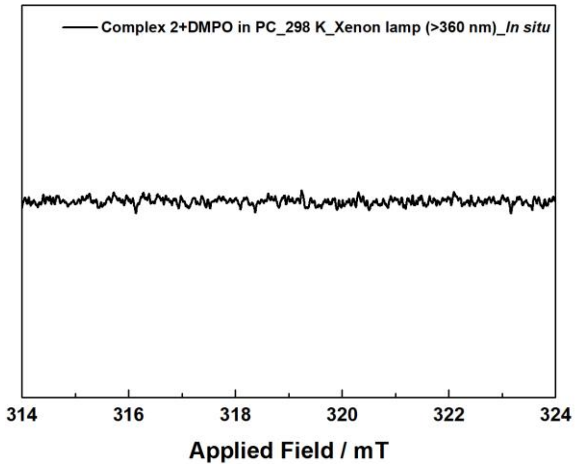

Supplementary Figure 24. EPR trapping with DMPO for 2. EPR spin trapping experiments with DMPO for **2** in PC under *in situ* irradiation with Xenon lamp (filter > 360 nm) in N_2 atmosphere.
